# Complete heart block associated with hepatitis A infection in a female child with fatal outcome

**DOI:** 10.1515/med-2024-0905

**Published:** 2024-02-05

**Authors:** Mansoor Ahmed, Haseena Naseer, Mohammad Ebad ur Rehman, Jawad Basit, Abdulqadir J. Nashwan, Mateen Arshad, Afnan Ahmad, Muhammad Asad

**Affiliations:** Department of Cardiology, Holy Family Hospital, Rawalpindi Medical University, Rawalpindi, Pakistan; Department of Pediatrics, Fauji Foundation Hospital, Foundation University Medical College, Islamabad, Pakistan; Nursing Department, Hamad Medical Corporation, Doha, P.O. Box 3050, Qatar; Department of Cardiology, Benazir Bhutto Hospital, Rawalpindi Medical University, Rawalpindi, Pakistan

**Keywords:** Hepatitis A virus, hepatitis, myocarditis, pacemaker, pediatrics

## Abstract

Hepatitis A virus (HAV) infection can cause extra-hepatic manifestations like myocarditis. An 8-year-old female with HAV infection presented with fever, abdominal pain, vomiting, and icterus. She developed viral myocarditis with complete AV dissociation on ECG and was treated with a temporary pacemaker, but her condition worsened, and she died. Hepatitis A viral infection can be associated with viral myocarditis and complete heart block that can lead to cardiogenic shock and death eventually.

## 1 Introduction

Hepatitis A virus (HAV) is a positive-strand RNA virus of the picornaviridae family. It may affect individuals at any age, from infancy to maturity. The majority of children afflicted are asymptomatic, but symptoms can range from mild to severe, with just 30% developing symptomatic hepatitis, whereas 80% of the adults exposed have clinical signs and symptoms of hepatitis [1]. Hepatitis A, unlike hepatitis B and C, does not cause chronic liver damage and normally cures in approximately 2 months [2]. The majority of viral hepatitis A transmission occurs through the feco-oral route. The most common form of transmission is food-borne transmission. Hepatitis A outbreaks have happened in the past across the world and are assumed to have a cyclical recurrence pattern [3]. Most common symptoms are fever, diarrhea, anorexia, abdominal pain, and jaundice. Adults are more often symptomatic than children and are more prone to develop complications.

Host immune response to HAV results in cellular death. An excessive immune response can lead to severe hepatitis and can rarely cause fulminant hepatitis and myocarditis [4,5]. Complications include cholestatic hepatitis, relapsing hepatitis, autoimmune hepatitis, fulminant hepatic failure, acute kidney injury, and other extrahepatic manifestations [1,2]. Rare extrahepatic manifestations include anemia, acute pancreatitis, neuritis, pleural and pericardial effusion, and myocarditis. The infection can lead to fulminant hepatitis and death in some patients [1]. After recovery, it provides a lifelong immunity.

There is no specific treatment for Hepatitis A. Supportive treatment is usually provided and the patient completely recovers from the disease most of the time within 2–6 months. In some patients, it can lead to fulminant hepatic failure, severe extrahepatic manifestation, and even death.

Hepatitis A leading to pericarditis, myocarditis, and pleural or pericardial effusion has been reported in the literature but is extremely rare [6,7]. Cases of complete heart block following infectious hepatitis have been published [8] and myocarditis following viral infection can lead to complete heart block [9,10]. Complete heart block secondary to amoebic hepatitis and measles are also reported in the literature [11,12], but we have not found any case report of complete heart block secondary to hepatitis A in the literature.

## 2 Case report

An 8-year old female child presented to emergency with complaints of high-grade fever for 7 days and yellow discoloration of the body for 3 days associated with vomiting and abdominal pain. On examination, she was pale, had a heart rate of 54 beats/min, a feeble pulse, a respiratory rate of 35/min. Normal lung sounds and normal heart sounds with bradycardia. She was afebrile on presentation; blood glucose level was 122 mg/dL. She was immediately admitted to the High Dependency Unit of Pediatrics and then to the Pediatric Intensive care Unit.

Lab investigations were carried out. Leukocytes were normal. The patient had increased alanine transaminase levels (995 U/L – Ref values: 5–25 U/L) and increased bilirubin levels 6.6 mg/dL. The patient had sodium levels at 126 mEq/L (Ref values: 135–145 mEq/L), potassium levels at 4.8 mEq/L (Ref values: 3.5–5.5 mEq/L), and calcium levels at 7.8 mg/dL (Ref values: 8.5–10.5 mg/dL). The patient had raised hepatitis A (HAV) IgM levels. She was primarily diagnosed as a case of acute viral hepatitis due to the HAV. Cardiac enzymes were found to be increased. creatine phosphokinase at 812 U/L (Ref values: 26–192 U/L), creatine kinase myocardial band at 122 U/L (Ref values: 5–25 U/L), and lactate dehydrogenase at 1,264 U/L (Ref values: 140–280 U/L) (Table 1). Supplemental oxygen was initiated. Electrolytes were corrected immediately, and intravenous calcium was administered. She was clinically suspected to be a case of viral myocarditis secondary to acute viral hepatitis.

**Table 1:** Clinical presentation

**A. Patient characteristics and clinical presentation on first DOA**	
Clinical examination	Clinical findings
Gender	Female
Age	8 years old
Fever	Afebrile
Heart rate	54 beats per min (Ref values: 67–103)
Respiratory rate	35 breaths/min (Ref values: 16–22)
Pulse	Feeble
Blood pressure	Not recordable
**B. Lab. investigations and findings on first DOA**	
Typhidot (for typhoid)	Negative
HAV IgM	Positive
HBV/HCV (ELISA)	Negative
ALT	995 U/L (Ref values: 5–25 U/L)
AST	549 U/L (Ref values: 10–40 U/L)
CPK	812 U/L (Ref values: 26–192 U/L)
CK-MB	122 U/L (Ref values: 5–25 U/L)
LDH	1,264 U/L (Ref values: 140–280 U/L)

Abbreviations: HAV, Hepatitis A virus; HBV, hepatitis B virus; HCV, hepatitis C virus; ALT, alanine transaminase; AST, aspartate aminotransferase; CPK, creatine phosphokinase; CK-MB, creatine kinase myocardial band; LDH, lactate dehydrogenase.

**Figure 1:**
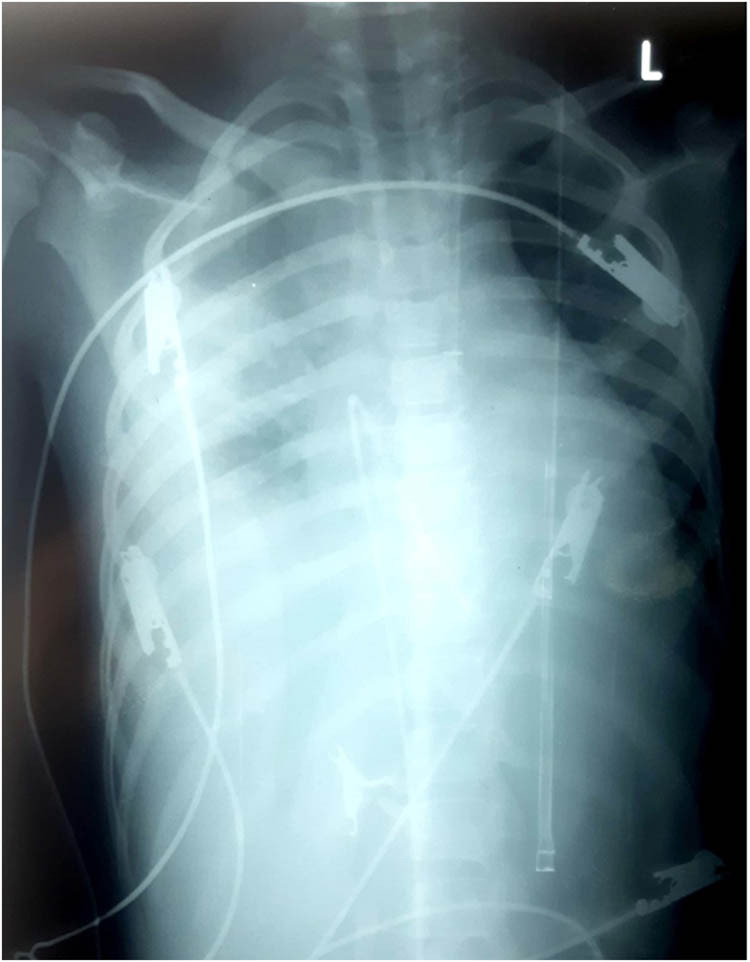
Chest X-ray showing pleural effusion and cardiomegaly.

**Figure 2:**
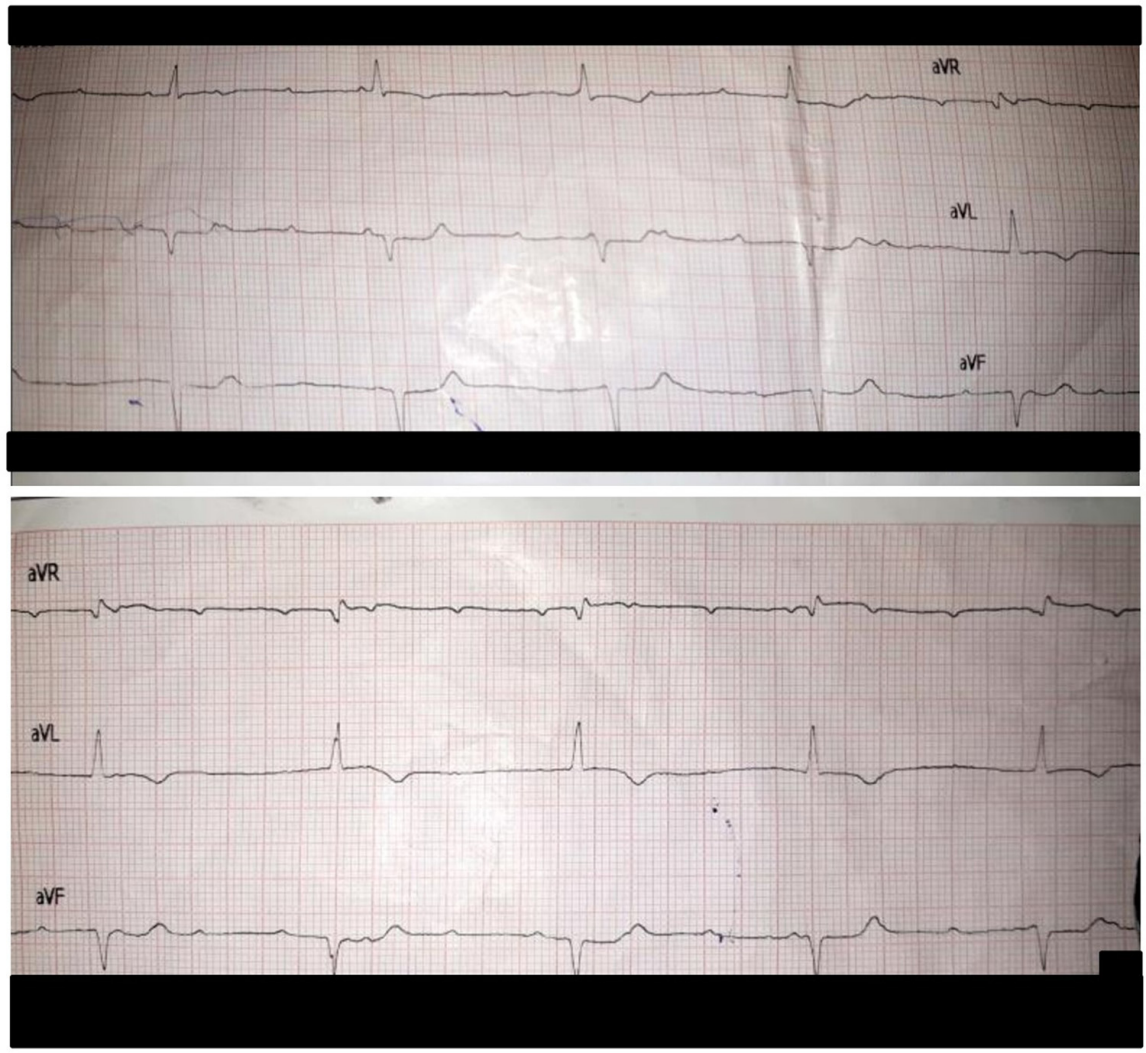
ECG (6-lead) showing complete AV dissociation.

Abdominal ultrasound showed pericholecystic edema, normal-sized liver, mild abdominal and pelvic ascites, and mild bilateral pleural effusion on chest USG. Chest X-ray was done which showed suspected pleural effusion on the right side with a blunt costophrenic angle and cardiomegaly (Figure 1). The fact that the chest X-ray was taken in a supine position can indeed have implications for the interpretation of the findings. The ECG shows upright P in inferior leads, positive in limb lead 1, isoelectric in augmented vector left, and inverted in augmented vector right. P–P interval is 480 ms (125 bpm). The ventricular complexes are narrow with R–R interval of 1,275 ms (47 bpm) and show complete dissociation from P waves suggesting third degree AV block. (Figure 2). The patient was managed in cardiology, and a temporary pacemaker (TPM) was placed immediately with initial settings programmed to 80 beats per minute (bpm) with an output of 70. The patient was medically managed with atropine and dopamine.

**Figure 3:**
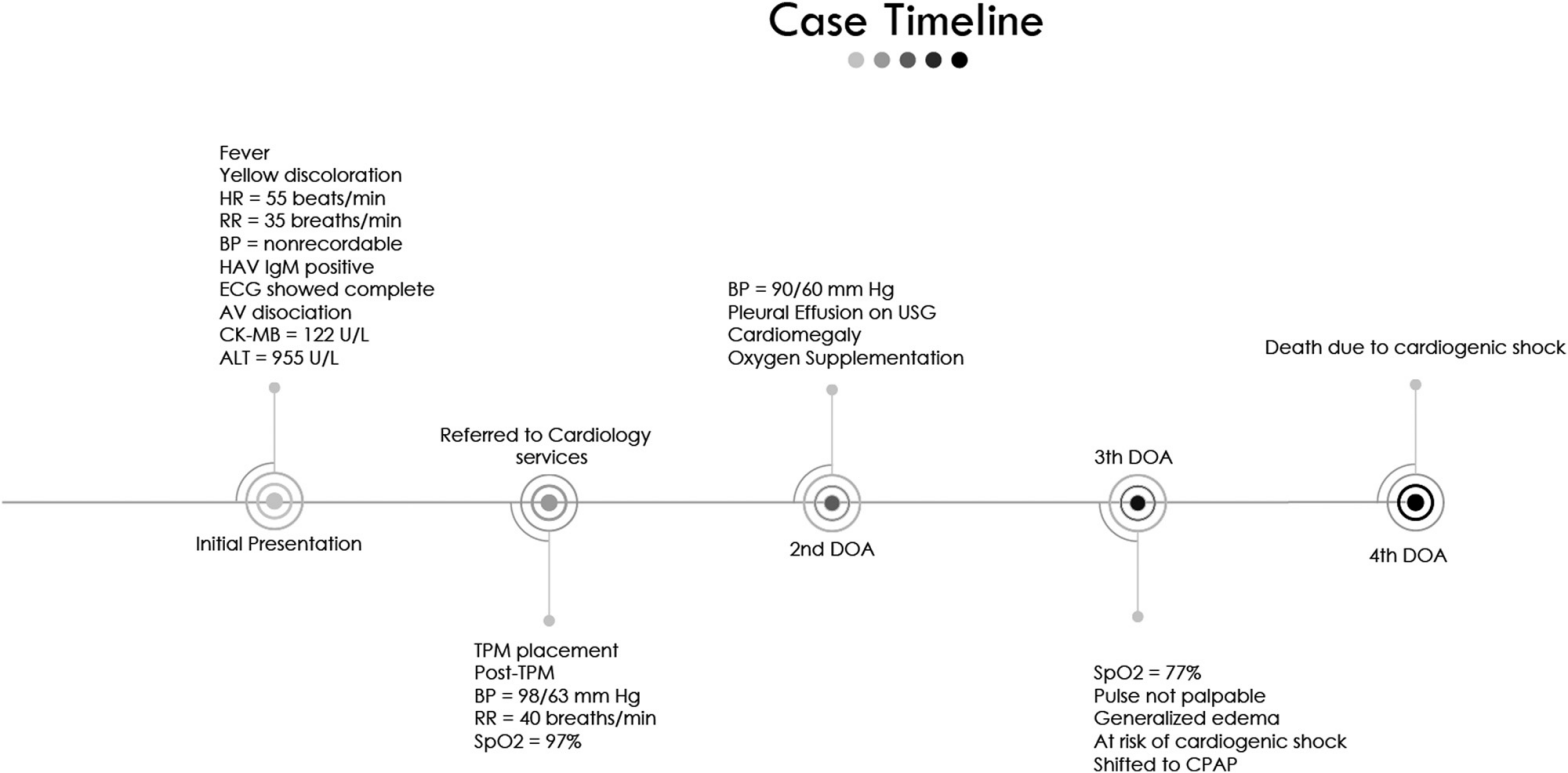
Case timeline.


On the third day of admission (DOA), oxygen saturation dropped further low to 77%. On examination, the patient was tachypneic, apprehensive, severely distressed, with nasal flaring, pulse was not palpable, and there was generalized puffiness along with pedal edema. There were bilateral crepitations in the lower zone; the abdomen was distended and tendered along with the firm liver. Arterial blood gases were ordered, which showed a blood pH of 7.49, HCO3 of 13.7 mEq/L, PCo2 of 17.9 mm Hg, and PO2 of 84 mm Hg. The patient was shifted to continuous positive airway pressure and was considered at risk of cardiogenic shock. The patient was managed with inotropes. Dobutamine, rivaroxaban in lower doses, and furosemide (0.5 mg/kg) were added to the treatment regimen. The patient died on the fourth DOA due to cardiogenic shock. The case timeline is shown in Figure 3.

**Informed consent:** A written informed consent was obtained from the parents to publish this report in accordance with the journal’s patient consent policy.

## 3 Discussion

The patient had no history of any cardiovascular disease and was admitted to the pediatric ICU as a case of acute viral hepatitis with secondary or associated cardiac involvement, with clinically diagnosed myocarditis and complete heart block. The patient was strongly suspected of complete heart block due to viral myocarditis secondary to acute viral hepatitis due to HAV, which is a very rare complication of HAV infection. The child had not been diagnosed with any congenital cardiac anomaly or congenital heart block. She had never been hospitalized before it. She never had any apparent cardiac risk and the family history was negative for any cardiomyopathy.

HAV infection is most of the time asymptomatic but can lead to fulminant hepatitis in some cases and some extra-hepatic manifestations like myocarditis [13]. Mechanism leading to myocarditis in HAV infection is not known but it is believed that the immune response to infected cell plays a central role in myocarditis [13]. The World Health Organization has reported that around 7,134 people died from hepatitis A worldwide in 2016 and the USA (CDC) reported around 12,474 cases of hepatitis A in 2018.

Myocarditis-induced heart block has been reported in the literature [14]. Viral pathogens are the leading cause of myocarditis. Myocarditis due to COVID-19 has been one of the dangerous complications of COVID-19 [15,16]. Other infectious causes of myocarditis reported in the literature include measles, amoebic hepatitis, Lyme disease, herpes, coxsackie, adenovirus, etc. [17,18]. Cases of viral myocarditis present with abnormal ECG and increased cardiac markers. Definite diagnostic investigations usually required in these cases are echocardiography, cardiac MRI, and endomyocardial biopsy, but the critical condition, need of a pacemaker, and limited resources often make it difficult to properly investigate a case of myocarditis. One should be cautious of myocarditis secondary to HAV infection and the patient must be screened with the help of cardiac markers in case the patient develops hypotension. Clinically suspected cases should be investigated at the earliest, and cases should be managed with supplemental oxygen and inotrope therapy [19]. TPM and automatic implantable cardioverter-defibrillator should be considered at the earliest. The use of a ventricular assist device is beneficial and should be offered considering the deteriorating condition of a patient and to those at risk of cardiogenic shock. Complete heart block due to myocarditis can be managed with pacemaker implantation but persistent low blood pressure even after installation of a pacemaker (as in this case) increases the risk of mortality in the child [20].

Hepatitis A is a vaccine preventable disease [21]. But it is also important to mention that Hepatitis A vaccine is not currently included in the national immunization program, Expanded Program on immunization, in Pakistan. Inflammatory cascade and autoantibodies against cardiac antigen in response to viral diseases have been studied in the past [5,23,24]. Although the efficacy of intravenous immunoglobulin (IVIG) in case of HAV complications has not been proven yet, there are reports of its successful use in case of HAV-induced myocarditis [5,22,24].

This case report highlights the rare complication of complete heart block secondary to viral myocarditis in a pediatric patient with acute viral hepatitis caused by the HAV. Despite the intervention (TPM), the patient’s condition continued to deteriorate, emphasizing the importance of maintaining a low threshold approach for monitoring vital signs in such cases. The importance of use of IVIG and specialized pediatric cardiac care, early recognition of cardiac complications in HAV infections, and the consideration of mechanical circulatory support are emphasized. Additionally, the non-inclusion of hepatitis A vaccine in the national immunization program is noteworthy, underscoring the potential for prevention of such complications.

## 4 Limitations

The cardiac MRI and myocardial biopsy are investigations of choice to confirm myocarditis [24]. Clinical picture of cardiomegaly and deranged cardiac enzymes without any ischemic manifestations raise clinical suspicion of myocarditis. We were not able to get a cardiac MRI of the patient because of the critical condition of the patient and the necessity of pacemaker; we had clinically diagnosed the patient as a case of viral myocarditis, leading to a complete heart block. We also want to highlight that ECHO was never performed in this case. The absence of an echocardiogram (2D-ECHO) in this case, despite the deteriorating medical condition, may restrict a comprehensive evaluation of cardiac function and potentially limit the understanding of underlying pathology. The clinical management of the patient was further constrained by the nonavailability of a pediatric cardiology specialist, which potentially impeded specialized expertise in managing complex pediatric cardiac conditions. IVIG was not administered in this case. Additionally, the lack of cardiac support services, such as access to advanced interventions or specialized equipment, may have contributed to inadequate treatment options and ultimately impacted patient outcomes, potentially leading to the unfortunate outcome of the patient’s death. Although the specific causal relationship in this case may be challenging to establish definitively, the temporal association between the positive hepatitis A status, the development of complete heart block, and the subsequent death raises the possibility of a potential link. Parents of the child did not consent to an autopsy.

## 5 Conclusion

Acute viral hepatitis caused by the HAV can be complicated by development of complete heart block secondary to viral myocarditis and may necessitate the installation of a TPM and can also lead to death. Specialized pediatric cardiac care is necessary to timely diagnose the condition. Close monitoring of the deteriorating cardiac condition of the child is important to prevent death. It is important to timely consider cardiac complications of symptomatic HAV infections and consider early shifting of patients to Mechanical Circulatory Support.

## References

[1] Jeong SH, Lee HS. Hepatitis A: clinical manifestations and management. Intervirology. 2010;53(1):15–9. 10.1159/000252779. 20068336

[2] Koff RS. Clinical manifestations and diagnosis of hepatitis A virus infection. Vaccine. 1992;10(Suppl 1):S15–7. 10.1016/0264-410x(92)90533-p. 1335649

[3] Daniels D, Grytdal S, Wasley A. Centers for Disease Control and Prevention (CDC). Surveillance for acute viral hepatitis – United States, 2007. MMWR Surveill Summ. 2009;58(3):1–27. 19478727

[4] Rezende G, Roque-Afonso AM, Samuel D, Gigou M, Nicand E, Ferre V, et al. Viral and clinical factors associated with the fulminant course of hepatitis A infection. Hepatology (Baltimore, Md). 2003;38(3):613–8. 10.1053/jhep.2003.50366. 12939587

[5] Botero V, García VH, Aristizabal AM, Gomez C, Perez P, Caicedo LA, et al. Hepatitis A, cardiomyopathy, aplastic anemia, and acute liver failure: A devastating scenario. Transpl Infect Dis. 2018;20:e12842. 10.1111/tid.12842 29359844

[6] Gordon SC, Patel AS, Veneri RJ, Keskey KA, Korotkin SM. Acute type A hepatitis presenting with hypotension, bradycardia, and sinus arrest. J Med Virol. 1989;28(4):219–22. 10.1002/jmv.1890280404. 2778446

[7] Atabek ME, Pirgon O. Unusual cardiac features in cholestatic hepatitis A in an adolescent: improvement with corticosteroid treatment. J Infect. 2007;54(2):e91–3. 10.1016/j.jinf.2006.05.001. 16769122

[8] Khongphatthanayothin A, Chotivitayatarakorn P, Benjacholamas V, Muangmingsuk S, Lertsupcharoen P, Thisyakorn C. Complete heart block in children at King Chulalongkorn Memorial Hospital. J Med Assoc Thai. 2001;84(Suppl 1):S111–7. 11529322

[9] Batra AS, Epstein D, Silka MJ. The clinical course of acquired complete heart block in children with acute myocarditis. Pediatr Cardiol. 2003;24(5):495–7. 10.1007/s00246-002-0402-2. 14627323

[10] Rawkins MD, Konstam GL. Complete heart-block associated with amoebic hepatitis; normal rhythm restored with emetine. Lancet. 1949;2(6569):152. 18146952

[11] Clark NS. Complete heart block in children; report of three cases possibly attributable to measles. Arch Dis Child. 1948;23(115):156–62. 10.1136/adc.23.115.156. PMC1988169 18885550

[12] Silver E, Pass RH, Kaufman S, Hordof AJ, Liberman L. Complete heart block due to Lyme carditis in two pediatric patients and a review of the literature. Congenit Heart Dis. 2007;2(5):338–41. 10.1111/j.1747-0803.2007.00122.x. 18377450

[13] Allen O, Edhi A, Hafeez A, Halalau A. A very rare complication of hepatitis A infection: Acute myocarditis-a case report with literature review. Case Rep Med. 2018;2018:3625139. Published 2018 Sep 13 10.1155/2018/3625139. PMC6158949 30302093

[14] Ogunbayo GO, Elayi S-C, Ha LD, Olorunfemi O, Elbadawi A, Saheed D, et al. Outcomes of heart block in myocarditis: A review of 31,760 patients. Heart, Lung Circulation. 2019;28(2):272–6. 10.1016/j.hlc.2017.12.005 29402690

[15] Fischer Q, Brillat-Savarin N, Ducrocq G, Ou P. Case report of an isolated myocarditis due to COVID-19 infection in a paediatric patient. Eur Heart J Case Rep. 2020;4(FI1):1–5. 10.1093/ehjcr/ytaa180. Published 2020 Jul 3. PMC7528934 33117957

[16] Ashok V, Loke WI. Case report: high-grade atrioventricular block in suspected COVID-19 myocarditis. Eur Heart J Case Rep. 2020;4(FI1):1–6. Published 2020 Aug 25 10.1093/ehjcr/ytaa248. PMC7499545 33089060

[17] Fung G, Luo H, Qiu Y, Yang D, McManus B. Myocarditis. Circ Res. 2016;118(3):496–514. 10.1161/CIRCRESAHA.115.306573. 26846643

[18] Yamamoto T, Kenzaka T, Matsumoto M, Nishio R, Kawasaki S, Akita H. A case report of myocarditis combined with hepatitis caused by herpes simplex virus. BMC Cardiovasc Disord. 2018;18(1):134. Published 2018 Jul 3. 10.1186/s12872-018-0869-2. PMC6029390 29970006

[19] Kang M, Chippa V, An J. Viral Myocarditis. [Updated 2022 Sep 6]. In StatPearls [Internet]. Treasure Island (FL): StatPearls Publishing; 2022. https://www.ncbi.nlm.nih.gov/books/NBK459259/.

[20] Chien SJ, Liang CD, Lin IC, Lin YJ, Huang CF. Myocarditis complicated by complete atrioventricular block: nine years’ experience in a medical center [published correction appears in Pediatr Neonatol. 2009 Feb;50(1):39]. Pediatr Neonatol. 2008;49(6):218–22. 10.1016/S1875-9572(09)60014-0. 19166118

[21] Herzog C, Van Herck K, Van Damme P. Hepatitis A vaccination and its immunological and epidemiological long-term effects – a review of the evidence. Hum Vaccin Immunother. 2021;17(5):1496–1519. 10.1080/21645515.2020.1819742. PMC8078665 33325760

[22] Özen M, Koçak G, Özgen Ü. Hepatitis A virus induced acute myocarditis. İnönü Üniversitesi Tıp Fakültesi Derg. 2006;13(1):47–9.

[23] Rose NR. Viral myocarditis. Curr Opin Rheumatol. 2016;28(4):383–9. 10.1097/BOR.0000000000000303. PMC4948180 27166925

[24] Law YM, Lal AK, Chen S, Čiháková D, Cooper LT, Deshpande S, et al. Diagnosis and management of myocarditis in children. Circulation. 2021;144(6):e123–e35. 10.1161/CIR.0000000000001001 34229446

